# KMC by surrogate can have an effect equal to KMC by mother in improving the nutritional behavior and arterial oxygen saturation of the preterm infant: results of a controlled randomized clinical trial

**DOI:** 10.1186/s12887-022-03316-z

**Published:** 2022-05-02

**Authors:** Mahboubeh Jamehdar, Roghaiyeh Nourizadeh, Aboulhassan Divband, Leila Valizadeh, Mohammadbagher Hosseini, Sevil Hakimi

**Affiliations:** 1 Student’s Research Committee School of Nursing and Midwifery, Tabriz University of Medical Science, Tabriz, Iran; 2 Faculty of Nursing and Midwifery, Tabriz University of Medical Science, Tabriz, Iran; 3 Faculty of medicine, Hormozgan University of Medical Science, Bandar Abbas, Iran; 4 Pediatric health Research Center, Tabriz University of Medical Science, Tabriz, Iran; 5 Faculty of Nursing and Midwifery, Research Center of Psychiatry and Behavioral Sciences, Tabriz University of Medical Science, Shariati Street, 5138947-, Tabriz, 977 Iran

**Keywords:** Kangaroo care, Nutrition behavioral, Physiologic function, Preterm newborn, O2 saturation

## Abstract

**Background:**

The aim of this study was to investigate the effect of kangaroo mother care (KMC) by mother and her surrogate on nutritional behavior and physiological function of preterm neonates.

**Method:**

This study was a randomized, controlled clinical trial conducted on 70 preterm infants admitted to the NICU. For the neonates of the intervention group, KMC was performed (by mother and surrogate) 3 times a day and the neonates of the control group received KMC by the mother 3 times a day for up to 4 days and 60 minutes each time. The primary outcome was to compare the effect of KMC by mother and surrogate on the feeding behavior measured by preterm infant breastfeeding behavior scale (PIBBS), and the secondary outcome was to compare the effect of KMC by mother and surrogate on physiological outcomes.

**Result:**

The score of the PIBBS in both groups increased significantly during 4 days, this difference was not significant between the groups. [Adjusted mean difference (95% Confidence interval), 0.66 (− 2.36 to 1.03), *P* = 0.438].

Within the group, among the physiological functions, only O2 saturation had significantly increased during the study. This increase, however, was not statistically different between the two groups. [Adjusted mean difference (95% Confidence interval), 0.102 (− 0.68 to 0.88), *P* = 0.761].

**Conclusion:**

When the mother is unable to provide this type of care, it can be provided by the surrogate that is as effective as the mother in improving arterial oxygen saturation and the feeding behavior of the preterm neonates.

**Trial registration:**

IRCT20150424021917N10. Registered 22/04/ 2020

## Background

Despite medical advances in the diagnosis and treatment of diseases, preterm birth is still a global health challenge both in developed and developing countries [1]. In 2016, prematurity was reported as the cause of 18% of deaths in children under 5, which is the most common cause of death in children under 5 worldwide [2]. Preterm neonates who survive, are at risk for many short-term and long-term complications [3]. Preterm birth accompanies with huge costs for the health system, and the families of these neonates often experience significant financial and psychological trouble [4]. The modern and complex process of caring for preterm neonates, which is called conventional care, leads to reduce neonates’ mortality and morbidity, however it needs to trained human resources and continues logistic. Therefore, the interventions cause to reduce mortality and care costs for preterm neonates are considered crucial [5].

Kangaroo mother care (KMC) method was early invented to care for preterm neonates in countries where incubator was not available and reliable [6]. This method can be provided by the mother, father and when the family cannot be with their neonate through the surrogate member. KMC prevents hypothermia. It improves sleep quality, provides physiological behavioral stabilization, growth and neurodevelopment of the neonate. KMC has positive effects on the psychological function of neonates, reducing parental stress as well as enhances attachment between mother and baby. This caring method creates a suitable thermal environment such as an incubator or even better, for the neonate [7]. Evidence shows that implementing KMC using quality improvement methods cause to save financial resources through reducing hospitalization, using antibiotics and formula in premature and low birth weight newborns [8].

Studies show that KMC has a remarkable positive effect on the success of the first breastfeeding and the duration of breastfeeding compared to routine care [9, 10] . Also, studies have not shown significant differences in stress and physiological responses following KMC and kangaroo father care [11, 12].

In many cases, mothers are unable to provide KMC. One of the most common causes of mothers’ inability to perform KMC and early initiation skin-to-skin contact (SSC) is cesarean section (CS). World Health Organization’s (WHO) recommendation to perform CS 10–15%, it is rising steadily in middle and high income countries [13].

Bahadori et al. showed that CS has remarkable increasing during recent years in Iran [14].

One of the solutions in these situations is doing the KMC by surrogate (including a family member or friend or members of the social network).

With regard to the need to do this type care by someone else of parents, it seems necessary to carry out this study and measure the objective and subjective neonatal consequences.

## Methods

### Study design

This study is aimed to compare the effect of KMC by mother and surrogate on feeding behavior and physiological functions of preterm neonates. We reported the result of test of hypothesis that O2 saturation, pulse and respiratory rate as well as feeding behavior in newborns gave KMC by surrogate are different with newborns who gave KMC by mother.

#### Participants

Participants in this study are preterm newborns.

#### Inclusion criteria

- neonates with the age of 32–35 weeks based on ultrasound performed before the 11th week of pregnancy;
- first or second pregnancy;
- the presence of a female surrogate to perform KMC.

#### Exclusion criteria

Using of CPAP or ventilator, existence of catheter in the umbilical vein, sepsis, interventricular hemorrhage, cleft lip and cleft palate, as well as skin rash;

- epilepsy among maternal or surrogate, new scar on a part of the mother’s or surrogate’s body, which is in direct contact with the neonate’s body and also the addiction of the mother or surrogate to any type of illicit drug were considered. This study performed on 70 premature neonates admitted to the neonatal intensive care unit of Khalij Fars Hospital in Bandar Abbas.

### Recruitment, randomization and data collection

In this study, sampling was purposeful. In this way, researcher chose the eligible neonates according to the inclusion criteria daily. Then the objectives, the advantages and risks of the study, if any, explained to mothers. Written informed consent was obtained from parents who agree to participate in the study, and a demographic / midwifery and neonatal profile questionnaire was completed. Participants was allocated randomly with block stratified by neonate age (32–33^+ 6^ and 34–35 weeks) with 4 and 6 block size with a ratio of 1: 1 to two KMC groups carried out by mother or by surrogate. The allocation sequence was determined using Random Allocation Software. For the allocation concealment, the type of intervention was written on a piece of paper and placed in sealed opaque envelopes, which were numbered in order, and the envelopes was opened by the non-involved person in the sampling, and the participant was placed in one of the two intervention or control groups. Data analysis stratergy is based on intention to treat.

### Data gathering tools

Demographic and neonatal characteristics questionnaire developed, with 24 questions including age (day) and gender of the neonate, neonate’s weight, gestation at birth (weeks), mother’s age, level of education, economic status, gravida, parity, type of pregnancy, mode of delivery, and surrogate relationship with mother (familial relationship, no familial relationship) “etc.”

Validity of social and individual characteristics questionnaires we used of qualitative content validity. The questionnaire will be sent to 10 reproductive health specialists and pediatrician and their views were be recorded and applied.

### Feeding behavior measurement

Preterm infant breastfeeding behavior scale (PIBBS) was developed by Nyqvist et.al (1999) to measure the feeding behavior of preterm neonates and its validity has been tested. The items of PIBBS evaluate six domains including: rooting, areolar grasp, longest duration of latching on, sucking, longest sucking burst, and swallowing (Table 1).

**Table 1:** The Preterm Infant Breastfeeding Behavior Scale (PIBBS)

Scale items	Maturational steps	Score
Rooting	Did not root	0
	Showed some rooting behavior Showed obvious rooting behavior	1 2
Areolar grasp	None, the mouth only touched the nipple	0
(how much of the	Part of the nipple	1
breast was inside	The whole nipple, not the areola	2
the baby’s mouth)	The nipple and some of the areola	3
Latched on and	Did not latch on at all so the mother felt it	0
fixed to the breast	Latched on for ≤5 min	1
	Latched on for 6–10 min Latched on for ≥11–15 min	2 3
Sucking	No sucking or licking	0
	Licking and tasting, but no sucking Single sucks, occasional short sucking bursts (2–9 sucks) Repeated short sucking bursts, occasional long bursts (≥10 sucks) Repeated (≥2) long sucking bursts	1 2 3 4
Longest sucking Burst	1–5 consecutive sucks 6–10 consecutive sucks 11–15 consecutive sucks 16–20 consecutive sucks 21–25 consecutive sucks ≥26–30 consecutive sucks	1 2 3 4 5 6
Swallowing	Swallowing was not noticed Occasional swallowing was noticed Repeated swallowing was noticed	0 1 2

A score between 0 and 2 is allocated to rooting, the score between 0 and 3 to areolar grasping, the score between 0 and 3 to longest duration of latching on, the score between 0 and 4 to sucking, score between 1 and 6 to longest sucking burst, and the score between 0 and 2 to swallowing; and a score of 20 is allocated to the neonate who is in the best feeding status [15].

The PIBBS questionnaire was translated by the forward and backward method after obtaining permission. The reliability of the PIBBS questionnaire was assessed through inter rater concordance, in this way the first author and a nurse staff completed a questionnaire for 10 neonates and the agreement was measured. The internal consistency of the instrument was measured by calculating Cronbach’s alpha. It was 0.791. Kappa Cohen agreement of the questionnaire in the first day was 0.72 and in the second day was 0.79.

### Physiological function measurement

Physiological functions include pulse and respiration rate, temperature as well as, arterial oxygen saturation (O2 saturation).

The researcher counted the number of pulse and respiration rate for 1 min. The neonate’s body temperature measured through axillary using a mercury thermometer.

For measurement of O2 saturation we used pulse oximetry. The pulse oximeter probe was attached to the neonate’s left thumb. Test-retest was done to check the reliability of pulse oximeter and thermometer. In this way that temperature, O2 saturation, and pulse and respiratory rate was measured for 10 neonates twice at 2-hour intervals, and the Intra-class correlation coefficient (ICC) was calculated. ICC All 4 variables mentioned were above 0.7 which is acceptable.

### Intervention

Ambient space included a comfortable chair, footrest, paravane, pillow and towel or blanket. Temperature of room was kept constant between 25 and 27 degrees. Participants washed their hands before contacting the newborn, put on a loose front shirt or hospital gown and then sat down a comfortable chair, as well as a pillow and a footrest was available for them to use. The newborn was then be placed between the mother’s or surrogate’s breasts. Newborn’s head was placed vertically or at an angle of 30–60 degrees, and back was covered with a blanket or cover. The mother or surrogate supported the baby while her hands are placed on neonate’s shoulders and back. Eye-to-eye contact with the neonate then made. Touch and voice contact began after the neonate has calmed down properly. For the control group, KMC was performed 3 times a day (once in each shift) for 4 days and 60 minutes each time by mother. For individuals of the intervention group, this care was done 3 times a day, once by the mother and twice by the surrogate, for 4 days and 60 minutes each time.

### Outcomes

Primary outcome of the study was to compare the mean score of PIBBS both intervention and control groups on the first, second, third and fourth days after the intervention completion with the control of feeding behavior score before the intervention using the PIBBS questionnaire. The PIBBS completed by a nurse who was not in the research team. For neonates of both groups, PIBBS was measured once before the intervention at the first day and then after the intervention completion for 4 days.

Secondary outcome of the study were the comparison of mean heart rate, respiratory rate, temperature, and arterial oxygen saturation between and within groups on the first, second, third and fourth days. The mentioned variables will be measured and recorded each time, once before and immediately after the intervention KMC for 4 days.

### Sample size

The sample size was determined based on Radzyminski’s study and using G-power software. Considering α = 0.05 and β = 0.2 with two tail tests and with considering PIBBS score m1 = 3.0 for the intervention group and assuming a 10% increase in the mean score in the control group m2 = 3.3 and SD1 = SD2 = 0.5, sample size of 35 people in each group and 70 neonates total sample size [16].

### Statistical analysis

Statistical analysis was carried out using SPSS sofware version 15. In assessment of normal distribution, the Shapiro-Wilk test was used. Demographic characteristics between two groups compared by t-test or Chi square. Physiologic function and feeding behaviour assessed by repeated measure Anova (RM Anova). All data had normal distribution.

## Results

**Fig. 1:**
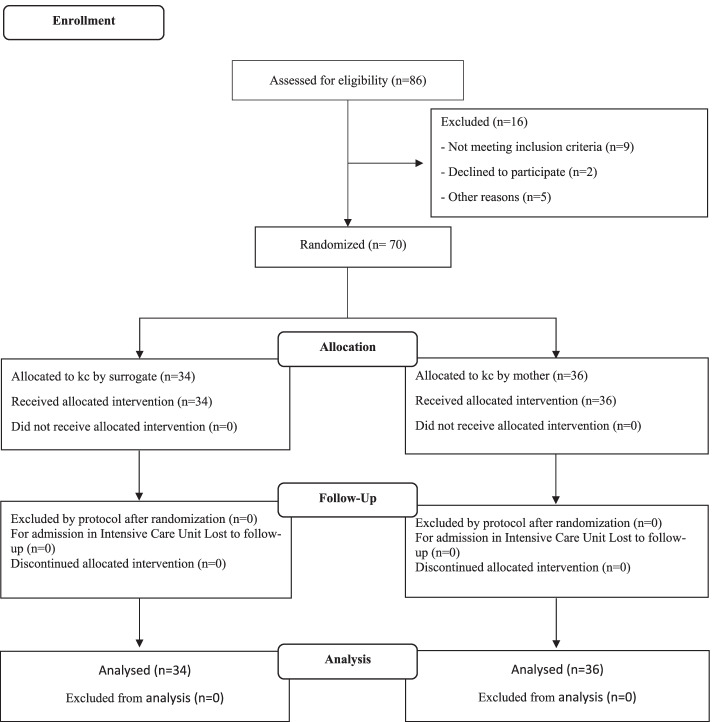
CONSORT study flow chart

This study was conducted on 70 preterm neonates from August 2020 to January 2021. A total of 86 neonate’s were assessed for eligibility. Sixteen (18.6%) did not meet the eligibility criteria (nine did not fulfill the inclusion criteria, two refused to participate and five discharged) (Fig. 1). The mean (standard deviation) age of mothers was 27.2 (0.6). The socio-demographic characteristics of the neonates/mothers participating in the study are given in (Table 2). The participants did not differ in terms of the delivery type, age and sex of the newborn, reception of breastfeeding counseling and use galactagogue. A half According to the results of the independent t-test, the mean score of PIBBS was not significantly different between the two groups in the first day before the intervention. The results of Greenhouse Geisser test indicated that the statistical model for RM Anova was suitable for comparing nutritional behavior and physiological function over time.

**Table 2:** Socio-demographic characteristics of mothers and infants based on the study groups

Variables	KMC by mother ***n*** = 36	KMC by surrogate ***n*** = 34	***P***
Gestational age	n(%)	n(%)	0.594
(32–33) Week	18 (50)	17 (50)	
(34–35) Week	18 (50)	17 (50)	
Gravida			
1	17 (47.2)	21 (61.8)	0.653
2	9	7	
> 2	10	6	
Newborn sex			
Male	22 (61.1)	19 (55.9)	0.420
Mode of delivery			
Caesarean	25 (69.4)	29 (85.3)	0.321
intended pregnancy	33 (91.7)	31 (91.2)	0.635
Breastfeeding counselling	12 (33.3)	9 (26.5)	0.358
Using of galactagogue	23 (63.9)	26 (76.5)	0.188

The results of RM Anova showed that the PIBBS score increased in both groups significantly during 4 days, but the difference was not significant between the two groups (Table 3). There was no difference between the two groups in terms of respiration rate, pulse, arterial oxygen saturation and temperature at the beginning of the study. According to the RM Anova, among the physiological parameters, only arterial oxygen saturation increased significantly over time. Additionally, there was no significant difference between the two groups in terms of the four intended variables (Table 4).

**Table 3:** Comparison of the newborns’ nutritional behavior between the two groups over time

	Day 1	Day 2	Day 3	Day 4	P*
**Groups**	**Mean (SD)**	**Mean (SD)**	**Mean (SD)**	**Mean (SD)**	**< 0.001**
KMC by mother n = 36	12.2 (4.7)	14.2 (4.2)	15.1 (3.6)	16.2 (3.7)	< 0.001
KMC by surrogate n = 34	12.3 (4.7)	13.9 (4.2)	14.1 (3.5)	14.7 (3.4)	0.021
Comparison groups		**AMD (95% CI)			
Mother-surrogate		0.66 (−2.36 to 1.03)			0.438

*Repeated measure ANOVA. Greenhouse – Geisser *P* value = 0.322, Partial Etta Square = 0.257

**Adjusted mean difference

**Table 4:** Comparison of the newborns’ physiological outcomes between the two groups over time

		Day 1	Day 2	Day 3	Day 4	***p***
**Groups**	**Physiological** **Parameters**	**Mean (SD)**	**Mean (SD)**	**Mean (SD)**	**Mean (SD)**	
KMC by mother n = 36	Respiration rate (per minute) Pulse rate (per minute) O2 saturation (%) Temperature (°C)	48.3 (4.6) 146.9 (11.5) 97.6 (1.7) 36.5 (0.3)	49.0 (4.5) 145.1 (8.5) 96.6 (4.8) 36.5 (0.2)	49.0 (4.0) 146.9 (9.7) 98.0 (1.2) 36.5 (0.3)	47.5 (4.4) 147.1 (8.7) 98.0 (1.2) 36.5 (0.2)	0.835 0.101 0.033 0.656
KMC by surrogate n = 34	Respiration rate (per minute) Pulse rate (per minute) O2 saturation (%) Temperature (°C)	48.0 (4.8) 150.1 (9.5) 97.6 (1.5) 36.5 (0.3)	46.9 (5.4) 149.2 (11.5) 97.6 (1.7) 36.5 (0.3)	47.1 (5.5) 150.6 (9.7) 97.1 (1.5) 36.4 (0.2)	47.2 (5.7) 150.8 (8.4) 97.5 (1.3) 36.5 (0.1)	0.625 0.153 0.043 0.361
Comparison groups		**AMD (95% CI)				
Mother-surrogate	Respiration rate (per minute) Pulse rate (per minute) O2 saturation (%) Temperature (°C)	0.68 (−2.09 to 0.73) 3.82 (−7.82 to 0.16) 0.102 (−0.68 to 0.88) 0.02 (−0.07 to 0.119)				0.341 0.06 0.761 0.641

*Repeated measure ANOVA

Respiration Greenhouse – Geisser *P* value = 0.425, Partial Etta Square = 0.351

Pulse rate Greenhouse – Geisser *P* value = 0.912, Partial Etta Square = 0.051

Temperature Greenhouse – Geisser *P* value = 0.751, Partial Etta Square = 0.005

O2 saturation Greenhouse – Geisser *P* value = 0.951, Partial Etta Square = 0.001

**Adjusted mean difference

## Discussion

This study was conducted on 70 preterm neonates to compare the effect of KMC by mother and surrogate on nutritional behavior and physiological functions. The results showed that the effect of KMC by the surrogate was not significantly different from that of KMC by the mother on the nutritional behavior and arterial oxygen saturation of the preterm newborn.

Breastfeeding is a two-way interaction between mother and newborn. KMC is one of the interventions which can affect mother-neonate interaction. After birth, newborn respond to the tactile, thermal, and olfactory stimuli of the mother’s body and are able to suck. During the SSC, the mother-neonate interaction improves, leading to feeding behaviors in the newborn [17].

SSC may enhance olfactory learning. The smell of breast milk is one of the stimulants of sucking, which stimulates the trigeminal and face nerves in the medulla oblongata and strengthens this innate behavior. The smell of breast milk improves the nutrition of preterm neonates and enables them to receive breast milk orally. The effect of stimulation with the smell of breast milk reduces the time required for passing from gavage to oral feeding. When the smell of breast milk reaches the neonate’s nose, salivary glands (amylase and lipase) begin to secrete, which in turn leads to the secretion of gastrointestinal glands, making sucking easier and more pleasant for the newborn [18].

There was no significant difference in this study between the two groups in terms of breastfeeding behavior score, meaning that KMC by the surrogate was as effective as by the mother in improving the feeding behavior of the preterm newborn. Given the fact that time is one of the effective factors in improving the score of infant feeding behavior, we modified the “time” effect. The test results showed that considering the effect of time, KMC by the surrogate is as effective as KMC by the mother on breastfeeding function.

During SSC, newborns experience the mother’s heartbeat, rhythmic breathing, warmth, and good posture, which help stabilize their temperature and heart rate, and reduce their respiratory rate [19].

The increase in O2 saturation following KMC may be due to the newborn’s gentle and comfortable contact with the mother and possibly a decrease in oxygen consumption. KMC stabilizes or increases temperature in a normal range. In other words, exposure of the newborn to SSC with the mother prevents heat loss. The rise of temperature is especially beneficial for the low birth weight and preterm infants with a tendency to hypothermia [20].

One of the most essential needs of neonates at birth is the maintenance of the body temperature as due to the lack of shivering mechanism, they are not able to produce heat which leads to a rapid decrease in their body temperature [21].

The rise of skin temperature in the newborns who have SSC with their mother immediately after birth is more obvious than the newborns who do not [22]. The mechanism of the effect of SSC on the neonate’s temperature is not fully known. It is said that touch, gentle pressure and, especially, the heat received by the newborn during the SSC activates the sensory nerves, leading to dilation of the skin vessels and increase of skin temperature [23]. Dilation of blood vessels or decreased vasoconstriction mediates the increase of the neonate’s body temperature. Additionally, reduction in vasoconstriction is the result of a decrease in sympathetic nerve tone. According to the findings, maternal temperature has a strong effect on the adjustment of the infant skin temperature. In fact, the neonate’s sensory system must be able to distinguish between very small changes in the mother’s temperature to adjust its temperature as accurately as the mother’s [22]. The result of a review showed that doing SCC by a father has few benefits including improvement of neonate’s temperature, physiologic functions as well as newborn’s behavior [12].

KMC has positive effects on stabilizing the respiration of preterm newborn. Kangaroo position (bending the body forward to 60 degree) allows preterm newborns to be below the upper part of the mother’s abdomen and increase the negative pressure below the diaphragm. Therefore, their respiration decreases, thereby contributing to their lung function. Improved heart rate is likely to be due to the newborn’s vertical posture and the mother’s soft and steady arms, which causes fast sleep and increases the body temperature of the preterm newborns. In general, KMC reduces the need for oxygen in preterm newborns as the upright position stabilizes respiration and improves O2saturation [24] .

In this study, 34 infants were included in the intervention group (KMC by surrogate) and 36 neonates in the control group (KMC by mother). Among the neonates of the intervention group, four neonates developed complications, that is, three neonates had restlessness during the care, and one infant had a recurrent drop in O2 saturation and underwent oxygen therapy. The care process was stopped for all four neonates and resumed the next day. In the control group, four newborns developed complications. One newborn had severe hypoxia (60) in the first care embrace and was, thus, immediately transferred to an incubator, oxygen was administered and visited by a physician. Necessary examinations were performed and the cause of the hypoxia was diagnosed to be anemia. Thus, packed cell was prescribed and the newborn recovered. Another newborn from the control group suffered from hypoxia on the fourth day of care to whom Oxygen was prescribed and examinations were performed, but no cause was found. Another newborn in this group had mild hypoxia on the second and third day of the care to whom oxygen was administered and no problems were seen after examinations. Srinath and colleagues in their study found that Kangaroo father care is as effective as KMC in improvement newborn’ physiologic function [11].

### Limitations

The subjects of this study were female surrogates and it was not possible to use a father or grandfather as a surrogate that was due to the lack of private space for performing KMC and cultural barriers.

## Conclusion

KMC is one of the cost-effective, easy and effective interventions for reducing many complications in premature newborns. Some mothers are unable to perform this type of postpartum care for reasons such as CS or labor-related morbidities. The results of this study showed that KMC by the surrogate can be as effective as the one presented by the mother in improving O2 saturation and nutritional behavior of the neonates.

Among the strengths of this study mention may be made of its design as a controlled clinical trial and randomization as well as concealment of allocation.

## Data Availability

The datasets used and/or analyzed during the current study available from the corresponding author on reasonable request.

## Abbreviations

KMC: Kangaroo mother care
WHO: World health organization
NICU: Neonatal intensive care unit
ANOVA test: Analysis of variance test
CPAP: Continuous positive airway pressure
PIBBS: Preterm infant breastfeeding behavior scale
IRCT: Iranian registry of clinical trials
CS: Cesarean section
